# Bacterial Dynamics and Their Influence on the Biogeochemical Cycles in a Subtropical Hypereutrophic Lake During the Rainy Season

**DOI:** 10.3389/fmicb.2022.832477

**Published:** 2022-04-05

**Authors:** Osiris Díaz-Torres, Ofelia Yadira Lugo-Melchor, José de Anda, Adriana Pacheco, Carlos Yebra-Montes, Misael Sebastián Gradilla-Hernández, Carolina Senés-Guerrero

**Affiliations:** ^1^Centro de Investigación y Asistencia en Tecnología y Diseño del Estado de Jalisco, A.C., Unidad de Servicios Analiticos y Metrologicos, Guadalajara, Mexico; ^2^Departamento de Tecnologia Ambiental, Centro de Investigación y Asistencia en Tecnología y Diseño del Estado de Jalisco, A.C., Zapopan, Mexico; ^3^Tecnologico de Monterrey, Escuela de Ingenieria y Ciencias, Monterrey, Mexico; ^4^ENES- León, Universidad Nacional Autónoma de Mexico, León, Mexico; ^5^Tecnologico de Monterrey, Escuela de Ingenieria y Ciencias, Zapopan, Mexico

**Keywords:** bacterial communities, fuctional properties, physicochemical and environmental parameters, subtropical lake, hypereutrophic lake, 16S rRNA gene sequencing

## Abstract

Lakes in subtropical regions are highly susceptible to eutrophication due to the heavy rainfall, which causes significant runoff of pollutants (e.g., nutrients) to reach surface waters, altering the water quality and influencing the microbial communities that regulate the biogeochemical cycles within these ecosystems. Lake Cajititlán is a shallow, subtropical, and endorheic lake in western Mexico. Nutrient pollution from agricultural activity and wastewater discharge have affected the lake’s water quality, leading the reservoir to a hypereutrophic state, resulting in episodes of fish mortality during the rainy season. This study investigated the temporal dynamics of bacterial communities within Lake Cajititlán and their genes associated with the nitrogen, phosphorus, sulfur, and carbon biogeochemical cycles during the rainy season, as well as the influences of physicochemical and environmental variables on such dynamics. Significant temporal variations were observed in the composition of bacterial communities, of which *Flavobacterium* and *Pseudomonas* were the dominant genera. The climatological parameters that were most correlated with the bacterial communities and their functional profiles were pH, DO, ORP, turbidity, TN, EC, NH_4_^+^, and NO_3_^–^. The bacterial communities displayed variations in their functional composition for nitrogen, phosphorus, and sulfur metabolisms during the sampling months. The bacterial communities within the lake are highly susceptible to nutrient loads and low DO levels during the rainy season. Bacterial communities had a higher relative abundance of genes associated with denitrification, nitrogen fixation, assimilatory sulfate reduction, cysteine, SOX system, and all phosphorus metabolic pathways. The results obtained here enrich our understanding of the bidirectional interactions between bacterial communities and major biogeochemical processes in eutrophic subtropical lakes.

## Introduction

One of the most important climatological attributes of tropical and subtropical regions is their heavy rainfall, which causes significant nutrient runoff from agricultural areas to surface waters. Additionally, the high temperatures and primary productivity that occur in these areas throughout the year make the superficial water sources highly susceptible to cultural eutrophication in comparison to temperate environments, where spring and summer are the more productive seasons (Cunha et al., 2013; Gradilla-Hernández et al., 2018, 2019, 2020). While rainfall and temperature can significantly alter the physical, chemical, and biological features of lakes in these regions, their water quality is the result of a complex set of multidirectional interactions among several factors, such as the natural and anthropogenic inputs of pollutants, environmental conditions (e.g., temperature and rainfall), the lake’s hydrodynamics, and *in situ* biological and chemical processes (e.g., microbial metabolism), among others (Niencheski and Windom, 1994). Furthermore, changes in the water quality of lakes lead to alterations in the microbial communities that regulate the biogeochemical processes within them, such as the sulfur, nitrogen, phosphorus, and carbon cycles (Liu et al., 2018; Wang et al., 2018; Yao et al., 2018); likewise, the metabolic activity of microorganisms greatly influences the water quality of lakes through different biochemical processes (Hupfer and Lewandowski, 2008).

The knowledge regarding the interactions between water quality and biogeochemical processes within lake ecosystems is still limited. Particularly, subtropical lakes pose an even greater challenge to understand the bacterial community structure and their corresponding biochemistry due to the rapid microbial changes, mainly caused by rainfall and superficial runoff (Díaz-Torres et al., 2021). Thus, there is a need to deepen the understanding on how bacterial metabolism and metabolic interactions drive biogeochemical transformations at the ecosystem scale (Newton et al., 2011; Graham et al., 2016). Metagenomic approaches have developed rapidly and have proven to be powerful for investigating microbial ecology and for linking microbial dynamics to biogeochemical processes (Mackelprang et al., 2011; Fierer et al., 2012; Llorens-Marès et al., 2015). Alternatively, phylogenetic analyses of communities by reconstruction of unobserved states (PICRUSt), employing 16S rRNA gene sequences and a reference genome database, have frequently been used to infer the functional profile of bacterial communities (Langille et al., 2013; Wilkinson et al., 2018).

Lake Cajititlán is a small, endorheic, subtropical, shallow lake located in the municipality of Tlajomulco de Zúniga in the state of Jalisco, Mexico (Díaz-Torres et al., 2021). Agriculture is the main economic activity in the basin, and most of the agricultural production takes place during the rainy season (rainfed agriculture), using large amounts of fertilizers and causing nutrient enrichment of the lake via surface runoff (de Anda et al., 2019). Furthermore, wastewater is discharged from three wastewater treatment plants (WWTPs) located along the reservoir’s shoreline, contributing to the nutrient enrichment as these facilities do not provide tertiary treatment to remove nutrients (de Anda et al., 2019). As a result of these sources of nutrient contamination, the lake’s water quality and ecological integrity have been significantly affected, bringing the reservoir to a hypereutrophic state, which has manifested through several episodes of fish mortality during the rainy season due to oxygen depletion (hypoxia/anoxia) at night, from 2013 to date (Gradilla-Hernández et al., 2018; de Anda et al., 2019).

Understanding the biogeochemical processes in hypoxic and anoxic lakes, such as Lake Cajititlán, will guide the efforts to improve water quality and to protect their aquatic life (Arora-Williams et al., 2018). However, most of the previous studies have failed to analyze the relationship between the taxonomic and functional variations of the microbial communities and the physicochemical and environmental variables affecting the water quality of lakes (Wilburn et al., 2019; Oyewusi et al., 2020; Ahmad et al., 2021). Additionally, most existing studies have been conducted in temperate and deep-water sources, with the majority focusing on one or two biogeochemical cycles (Staley et al., 2014; Koo et al., 2017; Ren et al., 2017; Arora-Williams et al., 2018; Kumar et al., 2018; Wilburn et al., 2019; Wu et al., 2019; Li et al., 2020; Oyewusi et al., 2020; Ahmad et al., 2021). Thus, the objective of this study was to analyze the dynamics of bacterial communities within Lake Cajititlán and their genes associated with the functional properties of the main biogeochemical cycles (nitrogen, phosphorus, sulfur, and carbon), as well as the influences of physicochemical and environmental variables on the taxonomic and functional composition of these communities during the rainy season (July–September). Based on our literature review, this is the first study focusing on bacterial diversity and its possible influence on biogeochemical cycles in a subtropical hypereutrophic lake during the rainy season.

## Methodology

### Study Area and Field Sampling

**FIGURE 1:**
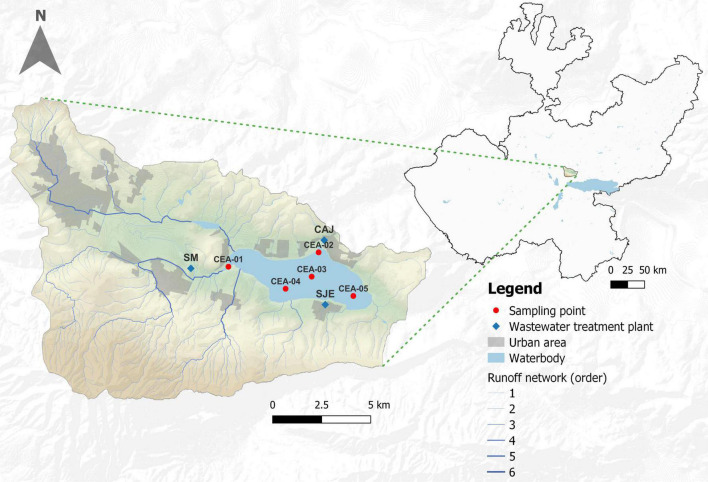
Location of the sampling points in Lake Cajititlán.

Lake Cajititlán is a subtropical endorheic lake located at an elevation of 1,550 m a.s.l in the central region of Jalisco, 25 km from the city of Guadalajara, which is the second largest metropolitan area in Mexico (Limón-Macias et al., 1983; Caro-Becerra et al., 2007; Figure 1). At maximum capacity, the surface area covers 1,744 hectares, the maximum storage volume is 70.89 Hm^3^, and the maximum depth is 5.4 m (de Anda et al., 2019). The hot-dry season in the region occurs during the months of February to May, the wet season occurs during June to September, and the cold-dry season occurs during October to January (Gradilla-Hernández et al., 2019). According to the monthly historical behavior (1998–2018; July–September) of precipitation, evaporation, and air temperature (AT) of Lake Cajititlán, July is the month which receives most rainfall (7.60 mm), followed by August (5.93 mm) and September (5.47 mm). Likewise, July presents the highest mean evaporation (5.39 mm) and the maximum temperature (26.51°C; CONAGUA Gobierno de México, 2021). The lowest mean minimum temperature was observed in August (12.84°C), followed by September (12.85°C) and July (13.26°C) (Supplementary Table 1; CONAGUA Gobierno de México, 2021). On the other hand, Díaz-Torres et al. (2021) reported an annual historical behavior (1998–2018) of TN:TP ratio and the ecosystem-specific water quality index (ES-WQI) for Lake Cajititlán, with July being the month that historically receives greater precipitation and, as a result, an intense runoff of pollutants to the lake, which annually shows the lowest values of the ES-WQI during July as well as the greater variations in the TN:TP ratio observed during the rainy season.

Sampling was conducted once a month during the rainy season (July–September) of 2018, as this study focuses on the changes in bacterial communities and functional composition during the rapid changes in rainfall and temperature that occur during this season. Water samples were collected from Lake Cajititlán at five sampling stations (CEA-1, CEA-2, CEA-3, CEA-4, and CEA-5) and at two monitoring depths (80 cm and interstitial) (Figure 1), using a Van Dorn water sampler, and placed in disinfected and washed 1-l plastic containers. Two replicates of each sample were obtained (2 L per sample), resulting in a total of 60 samples across all 3 months of the sampling period. Furthermore, water temperature (WT), dissolved oxygen (DO), pH, electrical conductivity (EC), ammonium (NH_4_^+^), nitrate (NO_3_^–^), turbidity, oxidation-reduction potential (ORP), blue-green algae (BGA-PC), and chlorophyll-*a* were measured once per month *in situ* at each site and sampling depth, using two multiparameter probes (6600 and 6829 V2 YSI^®^ a xylem brand, OH, United States) (YSI, 2009). The monthly EV, PREC, and AT data measured at the study site were retrieved from The National Water Commission of Mexico (CONAGUA by its Spanish acronym). The State’s Water Commission (CEA by its Spanish acronym) provided monthly data for total nitrogen (TN) and total phosphorus (TP); these measurements were made at the same sampling points and months as the other parameters, at a depth of 0.8 m. No variations were expected in the water quality parameters as Lake Cajititlán is a well-mixed lake due to the shear stress caused by the wind on the water surface (Gradilla-Hernández et al., 2019; Díaz-Torres et al., 2021).

### DNA Extraction, PCR, and Sequencing

Genomic DNA of the samples was extracted from two different pore sized cellulose nitrate filters to retain different microbial fractions. First, each replicate was filtered through a membrane with pore sizes ranging from 20 to 25 μm. The filtrate was then passed through a second membrane with a pore size of 0.45 μm. Each of these two filters was then cut into pieces with sterile scissors, and 100 mg of each was weighed and placed into a lysing matrix to perform a DNA extraction and purification of the samples using the FastDNA Spin Kit for Soil (MP Biomedicals, OH, United States), following the manufacturer’s protocol. The extracted DNA samples were quantified using a NanoDrop ND-1000 UV-Vis spectrophotometer (NanoDrop Technologies, Wilmington, DE). The 16S rDNA regions V3-V4 (∼550 bp, including indices and adapters) were amplified following the Illumina protocol for 16S Metagenomic Sequencing Library Preparation (Amplicon et al., 2013).

The amplified DNA was verified by 1.0% agarose gel electrophoresis in 1X TAE buffer. Sequencing libraries were cleaned using magnetic beads from the AM Pure XP kit (Beckman Coulter, IN, United States) and quantified with a Qubit 2.0 fluorometer (Invitrogen, Carlsbad, CA, United States). Finally, 96 samples were loaded onto the Illumina^®^ MiSeq sequencer (Illumina, San Diego, CA, United States) for 300-bp paired-end sequencing at the facilities of Tecnológico de Monterrey, Campus Monterrey.

### Sequence Analysis and Functional Gene Prediction

We used QIIME 2.0 v.2021.8 (Quantitative Insights into Microbial Ecology) to process raw 16S rRNA gene sequence data (Bolyen et al., 2019). Using DADA2 (p-trim-left 0, p-trunc-len 440 nts), chimeric, marginal sequence errors, and noisy sequences were filtered out while choosing amplicon sequence variations (ASVs) (Callahan et al., 2016). Following that, two characteristic tables [FeatureData(Sequence) and FeatureData(Taxonomy)] were generated with an identity level of 99% against the SILVA database version 138.1 for the 16S rRNA gene (Quast et al., 2012; Yilmaz et al., 2013). The q2-feature-classifier plugin was then used to assign taxonomy to representative sequences, using a trained Nave Bayes classifier (SILVA 138.1) for the 16S rRNA V3-V4 hypervariable region. Finally, using classify-sklearn, taxonomy classification was performed, and the designated sequences were archived using the trained classifier (Bolyen et al., 2019). To attach the corresponding taxonomy to the ASV table, taxon bar charts were created and downloaded in CVS format from view.qiime2.org.

The software, PICRUSt 2.0 v.2.4.1 was used for the predictive functional analysis of bacterial communities (Douglas et al., 2020). Representative sequences were first aligned with HMMER to determine functions (Eddy, 2011), and the alignment was placed in a reference tree (Czech et al., 2020) using SEPP and gappa. Then, using Castor, a hidden state prediction method, multiple copies of the 16S rRNA gene were normalized, and gene families were inferred (Louca and Doebeli, 2017). The MinPath was used to collapse the predicted gene families into MetaCyc pathways (Ye and Doak, 2009). Finally, the predicted metagenomes were classified into KEGG KOs. The KEGG database was used to identify KOs involved in carbon, nitrogen, and sulfur metabolism, as well as phosphorus cycling (Kanehisa et al., 2015, 2016)^1^.

### Statistical Analysis

All statistical analyses were carried out using the R v. 3.5.3 software unless otherwise stated (R Core Team, 2020). All bar graphs and line diagrams were built using the ggplot2 package (Wickham, 2009). The sequencing depth of the 16S rRNA genes was represented by a rarefaction curve generated using the rarefy function of the vegan package of the R software, which is based on Hurlbert’s (1971) formulation. The Heck et al. (1975) method was also used to calculate the standard errors (Oksanen et al., 2017). The DESeq2 package (Anders and Huber, 2010) was applied to normalize the number of reads obtained from 16S rRNA gene sequences. The Scale package was used to create bar graphs of the relative abundance of reads to examine and compare the information collected from reads at different months and sampling sites. Unclassified bacterial genera were labeled with “Un,” and the taxonomic level above was identified; taxa with proportions less than 0.01% were grouped as “other.”

Principal coordinate analysis (PCoA) was performed to observe microbial community differences (β-diversity) and to reveal functional differences in the metabolism of the biogeochemical cycles (carbon, nitrogen, phosphorus, and sulfur) between sampling points and months. Distances were based on Bray-Curtis dissimilarities using the vegan package and the cmdscale function. Additionally, an analysis of variance was performed using distance matrices (ADONIS) through the vegan package and the Adonis pairwise function to test the significance of spatial and temporal differences in the composition of the bacterial communities of Lake Cajititlán (Clarke, 1993; Anderson, 2001; Anderson et al., 2006).

Bar charts of relative abundance based on the metagenomic compositions of the KOs (relative abundance KOs) were made using the phyloseq and vegan packages (McMurdie and Holmes, 2013). To understand how environmental and physicochemical parameters influenced the abundance/composition of bacterial communities and their genes associated with biogeochemical cycle pathways, a table with the mean and standard deviation of each physicochemical and environmental variable by month of sampling was first created, followed by a one-way analysis of variance (ANOVA). Then, two redundancy analyses (RDA) were performed using the vegan package and ggord, a package for creating ordination plots (Beck and Mikryukov, 2021). The first one examined the correlation between microbial community variations and physicochemical and environmental parameters, whereas the second one related the functional composition of biogeochemical cycles and the physicochemical environmental parameters (Friedman, 1989; Press et al., 1992; Oksanen et al., 2017).

Finally, using the “corrplot” package, Mantel tests based on Spearman’s correlation between the abundant taxa of bacteria and the physicochemical and environmental factors were performed, and another correlation matrix was created to evaluate the relationships between the physicochemical and environmental factors with the metabolism (set of metabolic pathways) of the biogeochemical processes analyzed (Wei and Simko, 2021).

## Results

### Water Quality and Climatological Characteristics of Lake Cajititlán

The results of this study, which was conducted during the rainy season (July–September), revealed that most of the physicochemical parameters monitored showed significant temporal fluctuations when comparing the values reported for the three different months of the rainy season (Supplementary Table 1). The concentrations of NH_4_^+^ and NO_3_^–^ decreased over time (from July to September) whereas those of DO, ORP, and chlorophyll-*a* increased. Additionally, pH, turbidity, and BGA-PC all displayed similar and significant temporal patterns, with the highest levels observed in July, followed by a decrease in concentration in August and, finally, an increase in the last month (Supplementary Table 1). Ultimately, WT and CE presented similar temporal patterns, with the lowest values in July, an increase in August, and a decrease in September; however, only the differences observed for EC were statistically significant (Supplementary Table 1).

No significant differences were observed in the environmental parameters (EV, PREC, and AT) analyzed during the study period. However, the climatological parameter that displayed the greatest variation was PREC, and July was the month that displayed the highest amount of rain (Supplementary Table 1). Similarly, the highest EV and AT values were observed in July (immediately after the hot-dry season) (Supplementary Table 1).

### Bacterial Community Composition

**FIGURE 2:**
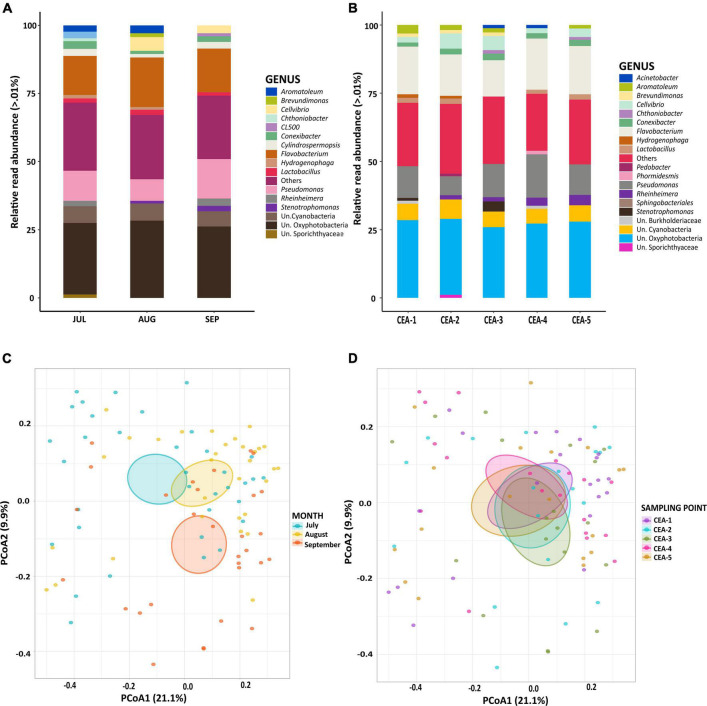
**(A)** Relative read abundance levels of bacterial genera by sampling month. **(B)** Relative read abundance levels of bacterial genera by sampling site. **(C)** PCoA of the bacterial communities to compare sampling months. **(D)** PCoA of the bacterial communities to compare sampling sites. Un.: Unknown.

The rarefaction curve of the 16S rRNA gene reads consistently reached their asymptotes, indicating sufficient sampling (Supplementary Figure 1). A total of 6,075,574 raw reads were obtained from all samples. Using the SILVA 138.1 reference database, a total of 4,135,845 reads (68%) were classified as bacteria. The composition of the bacterial communities was compared at the genus level to test for differences among sampling months (Jul, Aug, Sep) and sampling points (CEA-1, CEA-2, CEA-3, CEA-4, CEA-5). The genera *Flavobacterium* and *Pseudomonas* were most abundant in all months and at all sampling points. Similarly, the groups unknown (Un.) Oxyphotobacteria and Un. Cyanobacteria presented high abundances; however, these could not be classified to the genus level (Figures 2A,B). Significant temporal variations (ANOSIM *R* = 0.1233, *P* = 0.001) in the composition of bacterial communities were found over the study period when comparing the bacterial compositions within the samples taken during the different sampling months (ANOSIM *R* = 0.0024, *P* = 0.408; Figure 2C and Supplementary Table 2). However, spatial analysis showed that the composition of the bacterial communities was highly similar among all sampling sites as no significant differences were found (*p* > 0.05) (Figure 2D and Supplementary Table 3).

### Influence of Bacterial Communities on Biogeochemical Cycles

**FIGURE 3:**
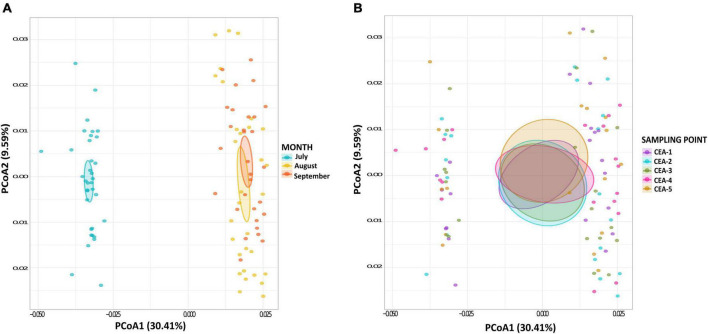
**(A)** PCoA of potential functions of all biogeochemical cycles by sampling month. **(B)** PCoA of potential functions of all biogeochemical cycles by sampling site.

**FIGURE 4:**
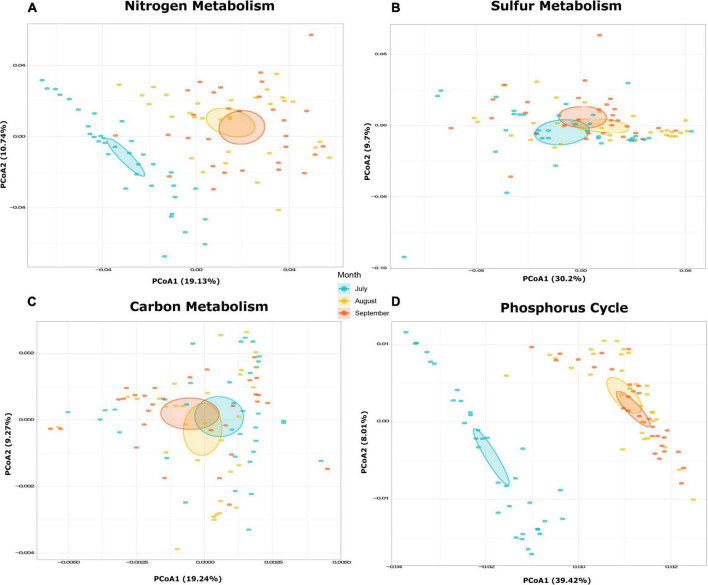
**(A)** Functional differences during July, August, and September. PCoA of potential function composition in terms of overall functions for **(A)** nitrogen metabolism, **(B)** sulfur metabolism, **(C)** carbon metabolism, and **(D)** phosphorus cycle.

A total of 1,416 KOs detected within the bacterial communities of Lake Cajititlán, which 26.84% (380 KOs) were related to the nitrogen, carbon, sulfur, and phosphorus biogeochemical cycles. The functional composition of the bacterial communities related to nitrogen, phosphorus, and sulfur metabolism was highly different among the sampling months and particularly different for the month of July (ANOSIM *R* = 0.003, *P* = 0.008; Figures 3A, 4 and Supplementary Table 4). Regarding the functional composition in terms of carbon metabolism, no temporal differences were observed (ANOSIM *R* = 0.0054, *P* = 0.29; Figures 3A, 4C and Supplementary Table 4). Moreover, no spatial variations in functional attributes were found regarding all biochemical cycles when comparing sampling sites, which was also the case for bacterial composition (Figure 3B and Supplementary Table 5).

Because there were no spatial significant differences in bacterial composition or functional attributes of biogeochemical cycling, specific metabolic pathways were only analyzed by sampling month and not by sampling site. A PCoA, based on the Bray-Curtis distances (Figure 4), was performed to examine the temporal behavior of the nitrogen and phosphorus metabolism. It revealed a temporal transition from the month of July to September, as the functional composition was significantly different between the month of July and the months of August and September (Figures 4A,C; nitrogen: ANOSIM *R* = 0.001, *P* = 0.001, phosphorus: ANOSIM *R* = 0.001, *P* = 0.001; Supplementary Table 4). The functional composition of the carbon and sulfur metabolism displayed a more stable behavior throughout the study period (Figures 4B,C). However, statistically significant temporal changes in the metabolic pathways of sulfur were observed (ANOSIM *R* = 0.022, *P* = 0.045), whereas no differences between the metabolic pathways were detected for carbon (ANOSIM *R* = 0.552, *P* = 0.067; Supplementary Table 4).

**FIGURE 5:**
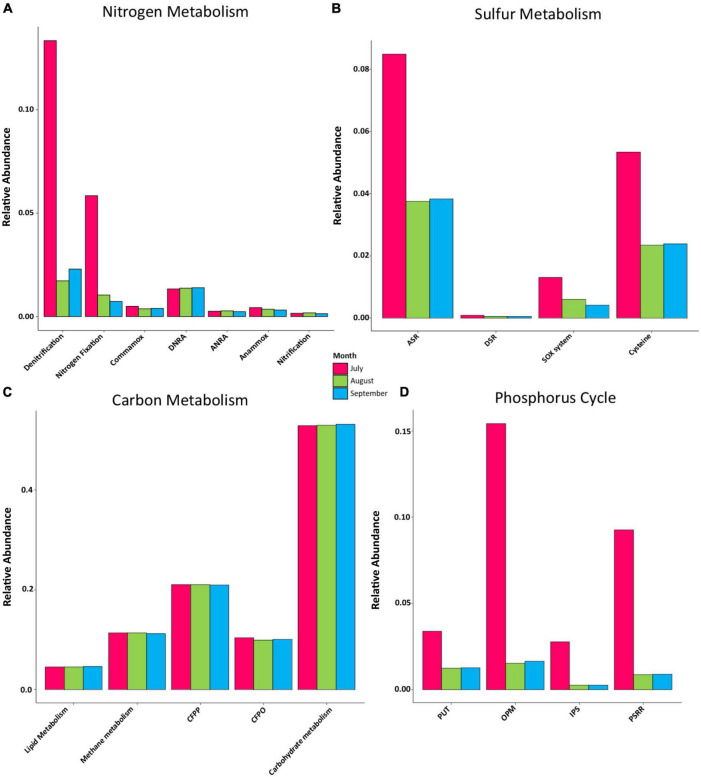
Relative abundance levels of genes associated with major pathways in **(A)** nitrogen metabolism, DNRA, Dissimilatory nitrate reduction to ammonia; ANRA, assimilatory nitrate reduction to ammonia. **(B)** Sulfur metabolism, ASR, assimilatory sulfate reduction; DSR, dissimilatory sulfate reduction; SOX system, sulfur oxidation system. **(C)** Central carbon metabolism, CFPP, carbon fixation pathways in prokaryotes; CFPO, carbon fixation in photosynthetic organisms. **(D)** Phosphorus cycle, PUT, P-uptake and transport; OPM, organic P-mineralization; IPS, inorganic P-Solubilization; PSRR, P-starvation response regulation.

Regarding the functional composition for nitrogen metabolism, 36 KOs were detected (ko00910 in the KEGG database). In this case, the bacterial functional composition displayed a significantly higher relative abundance of genes associated with the denitrification and the nitrogen fixation pathways, and July was the month with the highest gene abundance related to these pathways. Comammox and anammox were also slightly higher in July (compared to August and September), although these differences were not significant. The gene abundance levels related to dissimilatory nitrate reduction to ammonia (DNRA), assimilatory nitrate reduction to ammonia (ANRA), and nitrification processes were similar among the sampling months. Within these metabolic pathways, DNRA displayed the highest gene abundance during all sampling months (Figure 5A).

We found 23 KOs related to sulfur metabolism (ko00920 in the KEGG database). In July, all metabolic pathways displayed a higher relative gene abundance, with the assimilatory sulfate reduction (ASR) pathway displaying the highest gene abundance, followed by the cysteine, the sulfur oxidation system (SOX system), and the dissimilatory sulfate reduction (DSR) pathways. All metabolic pathways displayed similar abundance proportions in August and September, except for the SOX system, which was greatest in August (Figure 5B). According to the KEGG database, 287 KOs were related to the central carbon pathways in the analyzed samples (ko01200). The genes related to all bacterial carbon metabolic pathways displayed stable abundances during all sampling months, with carbohydrate metabolism being the most abundant one throughout the rainy season, followed by carbon fixation pathways in prokaryotes (CFPP), methane metabolism, carbon fixation in photosynthetic organisms (CFPO), and lipid metabolism (Figure 5C).

Overall, 34 KOs were found to be related to the phosphorus cycle. All phosphorus pathways examined were most abundant during July. The organic P-mineralization (OPM) pathway was the most abundant pathway, followed by P-starvation response regulation (PSRR), (PUT), and inorganic P-solubilization (IPS). Similar fractions were found between August and September for all metabolic pathways (Figure 5D).

### Physicochemical and Environmental Influences

Physicochemical and environmental factors are important determinants of microbial communities and their functional composition in Lake Cajititlán. The data matrix of the physicochemical and environmental parameters generated was used to explore the influence of these parameters on the bacterial communities’ functional composition during the study period (July, August, and September). Supplementary Table 1 shows the mean and standard deviations of the analyzed factors.

**FIGURE 6:**
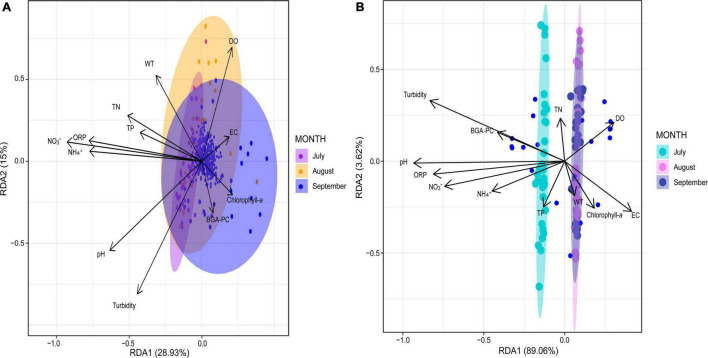
Biplot of the results of the redundancy analysis (RDA). **(A)** RDA regarding bacterial composition and physicochemical variables by sampling month. **(B)** RDA regarding functional composition and physicochemical variables by sampling month. Water temperature (WT), dissolved oxygen (DO), pH, electrical conductivity (EC), ammonium (NH_4_^+^), nitrate (NO_3_^–^), turbidity, oxidation-reduction potential (ORP), blue-green algae (BGA-PC).

The RDA indicated that the physicochemical parameters that were most correlated with the taxonomic composition during July and August were ORP, NH_4_^+^, NO_3_^–^, TN, pH, and WT, whereas chlorophyll-*a* was most correlated with the taxonomic composition in September (Figure 6A). The first three redundancy components explained 72.2% (RDA 1: 28.9%; RDA 2: 43.9%; RDA 3: 72.2%) of the overall variability of the physicochemical factors that influenced the temporal dynamics of the bacterial communities (Supplementary Table 6). The parameters that accounted for most of the bacterial variation in the first RDA component were pH, ORP, NH_4_^+^, and NO_3_^–^, all of which were negatively correlated with this component (Supplementary Table 7). The second component was highly correlated with DO (positive correlation) and turbidity (negative correlation), whereas pH (negative correlation) and TN (positive correlation) were the parameters most correlated with the third component (Supplementary Table 7). Positive correlations may suggest that microorganisms thrive under higher values of these (DO and TN) physicochemical parameters; on the other hand, the negative correlations (pH, ORP, NH_4_^+^, NO_3_^–^, Turbidity) imply that higher values of these parameters negatively affect the prevalence of the bacterial populations over the research period (Liu et al., 2014).

According to the RDA results, the physicochemical parameters that were most correlated with the functional composition of the bacterial communities of July were ORP, NH_4_^+^, NO_3_^–^, and pH, whereas TN, DO, WT, and chlorophyll-*a* were most correlated with the functional compositions for August and September (Figure 6B). The first three components explained 95.5% of the variability of functional composition (RDA 1: 89%; RDA 2: 92.6%, RDA 3: 95.5%; Supplementary Table 8); pH, ORP, and turbidity were the most correlated parameters with the first component, all of which displayed negative correlations. Turbidity was the variable that explained most of the variation in the second component, displaying a positive correlation. Finally, in the third component, NH_4_^+^ and NO_3_^–^ were the parameters that were most (positively) correlated with the genes of the bacterial communities involved in biogeochemical cycling (Supplementary Table 9). Positive correlations (NH_4_^+^ and NO_3_^–^) suggest that biogeochemical cycling activities are enhanced under these higher concentrations, whereas negatively correlated parameters (pH, ORP, and turbidity) may hinder biochemical cycling.

**FIGURE 7:**
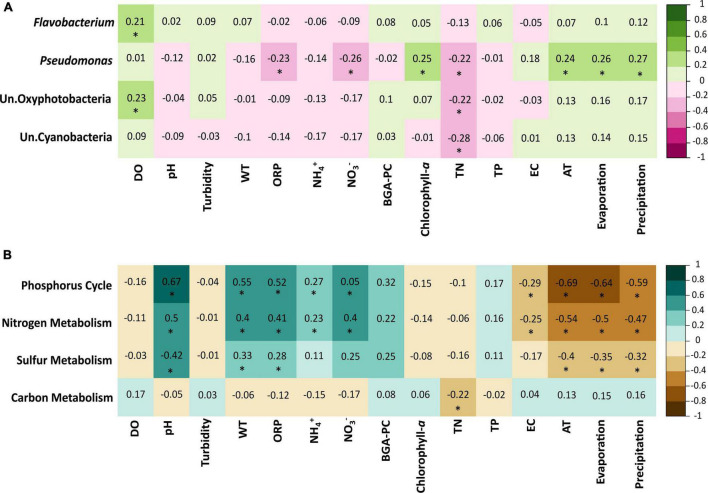
**(A)** Mantel tests based on Spearman correlations between most abundant taxa of bacteria and physicochemical factors. **(B)** Biogeochemical metabolism and physicochemical variables. Significant correlations (*p* < 0.05) are marked with an asterisk. Water temperature (WT), dissolved oxygen (DO), pH, electrical conductivity (EC), ammonium (NH_4_^+^), nitrate (NO_3_^–^), turbidity, oxidation-reduction potential (ORP), blue-green algae (BGA-PC), and air temperature (AT).

Mantel tests (Figure 7A) revealed that the composition and abundance of the genus *Flavobacterium* and the group Un. Oxyphotobacteria were positively correlated to OD, whereas the *Pseudomonas* genus was positively correlated to chlorophyll-*a* and all environmental variables (AT, EV, and PREC) but negatively correlated to ORP, NO_3_^–^, and TN, and the groups Un. Oxyphotobacteria and Un. Cyanobacteria were negatively correlated with TN. The correlation matrix between the biogeochemical metabolisms and the physicochemical and environmental variables (Figure 7B) show that nitrogen and phosphorus metabolisms were positively correlated with pH, turbidity, ORP, NH_4_^+^, NO_3_^–^, and BGA-PC and negatively with EC and all environmental parameters. Sulfur metabolism was significantly correlated with pH, turbidity, ORP, NO_3_^–^, and all environmental parameters. Finally, carbon metabolism was negatively correlated only with TN.

## Discussion

### Dynamics and Abundance of the Dominant Bacterial Taxa and Their Environmental and Physicochemical Influences

In recent decades, research on the dynamics of phytoplankton in lakes has increased, but there is still little knowledge regarding bacterial dynamics in such ecosystems, especially those in subtropical latitudes, despite the fact that many non-cultivable widely disseminated bacterial taxa have been discovered in freshwater environments (Malki et al., 2015; Sible et al., 2015; Barros et al., 2017; Guellati et al., 2017; Díaz-Torres et al., 2021). The heavy rainfall during the wet season causes rapid and significant variations in the water quality parameters in Lake Cajititlán (Gradilla-Hernández et al., 2019; Díaz-Torres et al., 2021). The study of bacterial communities within tropical and subtropical lakes represents a greater challenge due to the rapid changes that occur in physicochemical and environmental factors because of the heavy rainfall and increased flow events, which lead to increased runoff of allochthonous dissolved organic and inorganic matter as well as nutrients (Weyhenmeyer et al., 2004; Sadro and Melack, 2012; Stockwell et al., 2020; Díaz-Torres et al., 2021). Furthermore, variations in organic matter, dissolved solids, and nutrient concentrations can lead to alterations in the spatio-temporal dynamics of bacterial communities (Allgaier and Grossart, 2006; Grossart, 2010; Rösel et al., 2012).

In this study, no spatial differences were observed in the taxonomic composition of the bacterial community within Lake Cajititlán (Figure 2B). Shallow subtropical lakes, such as Lake Cajititlán, frequently exhibit a polymictic nature, with the total mixing of the water column occurring throughout the summer, mostly driven by precipitation and wind (Cavicchioli et al., 2019; Gradilla-Hernández et al., 2019). However, there were significant temporal variations in the composition of the bacterial communities among the sampling months (Figure 2A), which is consistent with a previous study focusing on the phytoplankton communities of Lake Cajititlán, which were found to be temporally, but not spatially, different (Díaz-Torres et al., 2021). This implies that bacterial communities may be influenced by phytoplankton diversity and abundance, as has been observed for other lakes, where changes in phytoplankton communities and environmental variables have been reported to influence the composition of bacterial communities and, together, phytoplankton and bacteria influence ecosystem-wide changes (Woodhouse et al., 2015; Ramanan et al., 2016).

The dominant bacterial genera found in Cajititlán were *Flavobacterium* and *Pseudomonas*, which were also predominant in prior investigations on bacterial communities in freshwater systems (Michaud et al., 2012; Shao et al., 2021). *Flavobacterium*, which is ubiquitous in freshwater environments (Groff and LaPatra, 2001), was most abundant during all sampling months and at all points (Figures 2A,B). This genus has previously been found in high abundance in eutrophic and hypertrophic freshwater ecosystems, such as Lake Cajititlán (Drury et al., 2013; Shao et al., 2021). It comprises over 30 species, of which *F. psychrophilum*, *F. columnare*, and *F. branchiophilum* can cause major diseases in fish populations within freshwater (Ilardi et al., 2009). Specifically, *F*. columnare is the cause of columnaris disease, a serious condition affecting numerous freshwater fish species worldwide (Starliper, 2011). Regarding Lake Cajititlán, there is substantial evidence that the massive events of fish death that occur yearly (since 2013) at the end of the wet season are a consequence of the lake’s eutrophication problem resulting in low oxygen (de Anda et al., 2019; Gradilla-Hernández et al., 2019; Díaz-Torres et al., 2021). Supporting this, during the sampling process for the present study, fish gasping at the surface and dead fish were observed floating on the surface (Supplementary Figure 2). Additionally, the production of microcystins by cyanobacteria has also been reported as a possible cause of the mass mortality of the fish in this lake (Díaz-Torres et al., 2021). Due to the findings of the present study, we hypothesize that pathogenic *Flavobacterium* species could be associated with the occurrence of massive fish death events in Lake Cajititlán, since it was the most abundant genus during the study period. However, culture-dependent, and pathological studies of fish stomachs are required to determine the causes of death and for a better characterization and taxonomic classification of the genus *Flavobacterium*, with the aim to determine which species of this group are present and whether they are pathogenic.

*Pseudomonas* was the second most dominant genus in Lake Cajititlán (Figures 2A,B). It is considered a cosmopolitan genus that has been found in a variety of habitats, including freshwater and terrestrial environments (Pearce et al., 2003; Saul et al., 2005), and includes opportunistic pathogens affecting humans, fish, and plants (Ferguson et al., 2004; Bleves et al., 2010). *Pseudomonas* is thought to promote the growth of the cyanobacteria that produce cyanotoxins by altering the phosphate exchange in their mucilage capsules (Jiang et al., 2007; Berg et al., 2008). Furthermore, in this study, the abundance of *Pseudomonas* was proportional to that of *Cylindrospermopsis* (Figure 2A), which are known to produce cyanotoxins (cylindrospermopsin, microcystin, and saxitoxin) and to have severe effects on water quality and aquatic ecology (Yilmaz and Phlips, 2011). A prior study on lake Cajititlán reported the presence of the toxin microcystin (Díaz-Torres et al., 2021), which has been suggested to give cyanotoxin degraders, such as *Pseudomonas*, a competitive edge by acting as a nutritional supplement (Kormas and Lymperopoulou, 2013; Mou et al., 2013; Zhu et al., 2014). Consequently, this toxin may contribute to the population growth of *Pseudomona*s in lake Cajititlán. This association has previously been reported for the eutrophic Lake Akersvannet in south Norway, where the highest concentration of cyanotoxins coincided with the highest diversity of potentially cyanotoxin-degrading species such as *Caulobacter*, *Novosphigobium*, *Sphingorhabdus*, *Paucibacter*, and *Pseudomonas* (Parulekar et al., 2017). In addition, it is well known that the composition of cyanobacterial blooms structures populations of heterotrophic bacteria, this is because cyanobacteria fix atmospheric nitrogen, and produce various dissolved organic compounds, providing heterotrophic bacteria with substrates for growth (Eiler and Bertilsson, 2004; Bagatini et al., 2014).

According to the RDA and mantel test, the parameters that were most correlated with the prevalence of bacterial communities during the study period were ORP, NH_4_^+^, NO_3_^–^, DO, turbidity, TN, and pH (Figures 6A, 7A and Supplementary Table 7). While ORP is a non-specific measurement reflective of all dissolved species in the medium, values ranging from + 100 to + 350 mV indicate a higher potential for the nitrification process, values ranging from + 50 to + 250 mV indicate higher values for carbonaceous biochemical oxygen demand, values ranging from + 25 to + 250 mV indicate higher biological phosphorus removal, and values ranging from + 50 to –50 mV indicate a higher denitrification potential (YSI, 2009). In this study, the mean ORP values during July, August, and September were 47.90, 66.18, and 101.78 mV, respectively (Supplementary Table 1). Therefore, all these processes may be enhanced in the lake during the different study months (July, August, and September) since the ORP values were measured within all these ranges.

Nutrient values (NH_4_^+^ and NO_3_^–^) were highest in July because of nutrient runoff from the agricultural lands surrounding the lake, where significant amounts of nutrients accumulated in the fields are washed off after the onset of the rainy season and as a result of the high evaporation rates (mean ∼9 mm) throughout the hot-dry season (February to May), and the decrease in nutrient concentrations throughout the study period (July to September) could be the consequence of a dilution process throughout the rainy season (Supplementary Table 1; Díaz-Torres et al., 2021). The parameters TN, NH_4_^+^, and NO_3_^–^ are crucial water quality parameters that drive the distribution of bacterial communities. Bacteria require a large amount of nitrogen for the synthesis of their main components, such as amino acids, pyrimidines and purines, NAD, and amino sugars. Furthermore, these organisms and archaea are the primary drivers responsible for converting different forms of N into chemical forms used by plants (NH_4_^+^and NO_3_^–^) (Reitzer, 1996; Wang et al., 2018). Some species of the genera *Pseudomonas* and *Cylindrospermopsis* observed in this study, as well as some species of the phylum Cyanobacteria (Figure 2A), could be involved in the nitrogen cycle pathways (Howarth et al., 1988; Crovadore et al., 2015; Paerl, 2017). The DO influences the abundances of functional genes involved in nitrogen and phosphorus cycling as well as redox reaction directions (Güven et al., 2005); it also has an impact on the growth of bacteria and the efficiency with which substances are degraded by them (Larson et al., 1981).

Alkaline pH levels (mean pH of 9.5) have already been reported to be caused by the natural geological conditions of the lake’s basin (Gradilla-Hernández et al., 2020). The pH plays a prominent role in determining the metabolisms that appear in bacterial communities within specific ecological systems; it also influences the composition of bacterial communities; e.g., *Flavobacterium* is strongly positively correlated with this parameter, as shown in this study (Figure 7A) and as reported previously (Garcia et al., 2013; Wang et al., 2021). Finally, turbidity was another important variable correlated with the bacterial communities of Lake Cajititlán; this parameter is seasonally dependent and influenced by factors such as the presence of phytoplankton, suspended sediments, and effluent discharges. Therefore, the bacterial communities within the phytoplankton are reflected by this parameter and/or the nutrients that are part of the suspended materials that affect the prevalence of photosynthetic bacteria within the lake (Myint and Walker, 2002; Harsha and Malammanavar, 2004).

### Biogeochemical Cycles Linked to Bacterial Communities and Their Environmental and Physicochemical Influences

Previous research on eutrophic freshwater lakes has focused on the dynamics of phosphorus in shallow lake sediments (Søndergaard et al., 2003; Lukkari et al., 2008; Hayakawa et al., 2015). Based on the results, in the sediment of shallow lakes, numerous highly dynamic processes with substantial effects on the total phosphorus budget and lake water quality occur (Søndergaard et al., 2003). Additionally, other studies have reported that human activities influence nitrogen removal from lakes, the vertical distribution of phosphorus in shallow estuaries, and the retention and internal loading of phosphorus in shallow and eutrophic lakes; however, few studies have focused on the spatial and temporal variations of nutrients in water of a shallow, eutrophic, and subtropical lakes (Søndergaard et al., 2001; Zhang et al., 2007; Lukkari et al., 2008; Finlay et al., 2013; Hayakawa et al., 2015). In the case of Lake Cajititlán, the water quality and the composition of the phytoplankton community shift significantly during the wet season (Díaz-Torres et al., 2021), making it crucial to understand the contribution of bacterial communities to the larger-scale ecological biogeochemical processes as well as the mechanisms that lead to the assembly of bacterial communities during the rainy season in such ecosystems (Wu et al., 2019).

According to the RDA and the mantel test results, the physicochemical and environmental parameters that were most correlated with the functional composition of the bacterial communities were pH, ORP, turbidity, TN, EC, NH_4_^+^, and NO_3_^–^ (Figures 6B, 7B and Supplementary Table 9). Except for EC, all these variables were also the most correlated with the prevalence of bacterial communities over the research period. The EC is a multifactorial parameter influenced by several factors such as the geology of the basin, wastewater discharges, runoff from diffuse sources, atmospheric inputs, evaporation rates, and some types of bacterial metabolism, among others (Pal et al., 2015). In Lake Cajititlán, the high concentration of ions can largely be explained by anthropogenic contamination, which can be somewhat beneficial to bacterial growth (Torres et al., 2008; de Anda et al., 2019). As previously stated, the bacteria most prevalent in this study influenced the biogeochemical cycles analyzed, as evidenced by their correlation with the majority of the physicochemical parameters observed in both correlation analyses (taxonomy and functional).

### Nitrogen Metabolism

Nitrogen exists in a variety of chemical forms, such as NO_3_^–^ and NH_3_^+^, which are cycled through several biogeochemical processes (Ollivier et al., 2011), including four reduction pathways (DNRA, denitrification, ANRA, and nitrogen fixation) and three oxidation pathways (anammox, nitrification, and comammox) (Lamba et al., 2017; Mehrani et al., 2021). Denitrification and anammox are processes that remove nitrogen from aquatic habitats (Ward et al., 2009). Denitrification is the principal biological process in aquatic ecosystems through which nitrate is converted into dinitrogen and nitrous oxide (Bernhard, 2010), whereas anammox is a key pathway for converting nitrite and ammonia to dinitrogen (Kuypers et al., 2005). In this study, the denitrification pathway displayed the highest abundance of genes during July, indicating a significant potential for nitrogen removal, which is consistent with the highest levels of NO_3_^–^ as well as the lowest levels of ORP observed in the same month which could enhanced denitrification process in Lake Cajititlán (Sigman and Casciotti, 2001; Włodarczyk et al., 2003; Higgins, 2013; Vizcaíno-Rodríguez et al., 2018; Díaz-Torres et al., 2021; Figure 5 and Supplementary Table 1). These water quality features interact with microorganisms in a complex way on a microscale level, resulting in spatial and temporal variability in the denitrification rates (Parkin et al., 1987; Svensson et al., 1991; Włodarczyk, 2000). It has also been reported that microbial respiration within the sediments can cause anoxic environments, further promoting denitrification (Lv et al., 2017). Thus, the denitrification pathway was also enhanced by the extremely low DO levels (2.83 mg/L) displayed in July in Lake Cajititlán.

The functional composition also displayed a high abundance of genes associated with nitrogen fixation during July (Figure 5A). Nitrogen fixation and denitrification can co-occur in sediments through heterotrophic nitrogen fixation (Newell et al., 2016). However, nitrogen fixation is believed to occur predominantly at the surface of water bodies through the activity of diazotrophic phototrophs, such as cyanobacteria, which are the most important nitrogen fixers in aquatic environments (Zehr et al., 2001; Montoya et al., 2004; Capone et al., 2005). Since no diazotrophic heterotrophic bacteria were found at high abundance in Lake Cajititlán, it may be assumed that nitrogen fixation was mostly performed by *Cylindrospermopsis* species, which are diazotrophic phototrophs (Howarth et al., 1988; Paerl, 2017). Furthermore, it is likely that the nitrogen-fixing prokaryotic bacteria contributed to the massive algal blooms that have occurred in Lake Cajititlán in recent years due to the symbiotic relationship between nitrogen-fixing prokaryotic bacteria and algae, as the latter are incapable of fixing atmospheric nitrogen, which is required for their growth (Thompson et al., 2012).

Regarding the remaining nitrogen metabolism pathways (comammox, DNRA, ANRA, anammox, nitrification), no temporal differences were observed. However, a higher relative abundance of DNRA-associated genes was observed during all months of the study in comparison with other pathways (Figure 5A), suggesting a stable functionality of the bacterial communities in the water column to reduce nitrate to ammonia. The metabolic pathways ANRA, DNRA, and denitrification use nitrate. The first pathway is expected under aerobic conditions when reduced nitrogen is limited, whereas the other two dissimilatory pathways are expected when oxygen is limited (anaerobic conditions), and their respective reduced products (NH_4_^+^ and N_2_ + N_2_O, respectively) are produced in relatively greater quantities because these processes are linked to electron transport (Teje et al., 1981; Henson et al., 2017). Based on our OD measurements (Supplementary Table 1), it can be inferred that the predominant nitrogen pathways in Lake Cajititlán are those that occur under anaerobic conditions (denitrification, nitrogen fixation, and DNRA), which is consistent with extensively reported massive phytoplankton blooms occurring in the lake (Gradilla-Hernández et al., 2019), which can produce high DO levels and biomass growth during the day (coupled with a reduction in light penetration and photosynthetic activity) and anoxic or hypoxic conditions at night as a result of their respiration (Smith and Schindler, 2009).

### Carbon Metabolism

During the 3 sampling months, the functional composition related to the carbohydrate metabolism pathway was most abundant within all carbon metabolism pathways, followed by CFPP, methane metabolism, CFPO, and lipid metabolism. However, no temporal differences were observed among them (Figure 5C).

Microorganisms play an important role in the production, transformation, and mineralization of organic matter (Powlson et al., 2001; Amalfitano et al., 2007). As a result, the functional composition of bacterial communities is largely determined by fluctuations in the organic matter composition of the water column as well as the bioavailability of the different chemical organic forms (Hoostal and Bouzat, 2007; Wang et al., 2018). Organic matter comprises carbohydrates, proteins, lipids, lignins, and other compounds (Thurman, 2012). Besides the organic matter entering the lake through the WWTP effluents, organic matter is a heterogeneous mixture that contains allochthonous materials from terrestrial ecosystems, such as soil and plant litter, as well as autochthonous materials from primary producers (Finlay, 2001; Mosisch et al., 2001; Carpenter et al., 2005; Cole et al., 2011; Solomon et al., 2011). Thus, the high organic matter concentrations in the water column of Lake Cajititlán largely influence the most abundant pathway for carbon metabolism, which is carbohydrate metabolism (Figure 5C; Vizcaíno-Rodríguez et al., 2018). The raw or partially treated wastewater discharged into Lake Cajititlán by three treatment facilities is a major source of energy for the reservoir’s bacterial populations. Furthermore, especially during the rainy season, the organic matter pollution is exacerbated because the capacity of the treatment plants is surpassed as the raw wastewater is combined with rainfall and an overflow wastewater bypass is implemented at the treatment plants, discharging the wastewater and rainfall mixture directly into the lake (de Anda et al., 2019; Gradilla-Hernández et al., 2019).

Both ORP and DO are critical water quality parameter for the carbon cycle, as reducing conditions and low DO levels can enhance methane synthesis, whereas methane-oxidizing and sulfate-reducing bacteria contribute to carbon fixation (Kellermann et al., 2012; He et al., 2015; Itoh et al., 2015). Sulfate-reducing bacteria (SRB) are anaerobic microorganisms that use sulfate as an electron acceptor during the dissimilatory sulfate reduction process (DSR) (Simon and Kroneck, 2013). In this research, it can be inferred that these bacteria could not influence the CFPO pathway (carbon fixation pathways in prokaryotes) since no differences were observed in the abundance of this pathway during the sampling months. In addition, the DSR pathway was least abundant in sulfur metabolism (Figure 5B). In contrast, the functional abundance of the methane metabolism was enhanced with low DO levels (Figure 5C and Supplementary Table 1), which leads us to infer that the methane-oxidizing bacteria and/or archaea governed this pathway since the SRB bacterial activity would have been reflected by the DSR pathway. It is also widely reported that SRB can depend on methanogens that scavenge hydrogen and acetate to convert organic compounds to methane. Under low DO concentrations, SRB and methanogens do not compete and rather cooperate in the degradation of organic matter (Plugge et al., 2011), which may occur in this lake during the rainy season.

### Phosphorus Cycle

Anthropogenic activities have altered the phosphorus cycle in aquatic environments (Kononova and Nesmeyanova, 2002; White and Metcalf, 2007). For example, the intensive use of highly water-soluble phosphorus fertilizers has contributed to the eutrophication, and hypoxic conditions, of surface waterways across the world (Haque, 2021). Organophosphates are widely used in the chemical industry as detergents, antifreeze compounds, and pesticides and can also be found in a wide variety of antibiotics of microbial origin, which are not generally treated properly in municipal WWTP and thus contribute to nutrient accumulation in water sources (Hayes et al., 2000; White and Metcalf, 2007). Bacteria play an important role in phosphorus cycling in water sources, and in this study, the findings indicate that in July, all phosphorus metabolic pathways were more abundant compared to August and September. The most abundant pathway was organic OPM, followed by PSRR, PUT, and IPS (Figure 5D). Plants and microorganisms obtain phosphorus from inorganic sources rather than organic ones, and the process by which organic phosphorus is converted into organic phosphate is call mineralization (Vance, 2001). Phosphate esters are the most abundant organophosphorus compounds in the biosphere and the easiest to mineralize due to the low energy demand in the production of the exoenzymes (phosphatases, phosphonatases, and C-P lyases) required, which are produced by several microorganisms of the genera *Bacillus*, *Pseudomonas*, *Azotobacter*, *Rhodococcus*, *Serratia*, *Bradyrhizobium*, *Salmonella*, and *Thiobacillus*, among others (Kolowith et al., 2001; Kononova and Nesmeyanova, 2002; Postma et al., 2010; Babalola and Glick, 2012; Jahan et al., 2013; David et al., 2014). In this research, *Pseudomonas* were the second most abundant bacterial genus and could significantly contribute to phosphorus mineralization in Lake Cajititlán (Figure 2A) since it produces phosphatase under conditions of low P availability, providing it with an advantage over other phosphorus-mineralizing microorganisms (Thaller et al., 1995; Babalola and Glick, 2012).

Phosphorous limitation triggers a P starvation response in aquatic environments, which can accelerate nutrient uptake by microorganisms and plants, resulting in a reduction in the TP content (Grossman and Aksoy, 2015; Slocombe et al., 2020). A previous study reported the annual behavior of the TN:TP ratio within Lake Cajititlán over the past 21 years (1998–2019) and showed that nutrient limitation in this reservoir can shift during the rainy season. A 10-year data set study revealed that Lake Cajititlán is phosphorus limited during the beginning of the rainy season (June-July) and at the end of the rainy season (September) but nitrogen-limited in August (Díaz-Torres et al., 2021), and these shifts are driven by the rainy season. The authors of this study discussed how these variations in the limiting nutrient could be attributed to intensive fertilizer runoff at the start of the rainy season, which increases the TN: TP ratio during June and July, which is then decreased in August due to increased nitrogenous compound consumption by growing phytoplankton communities. These authors also explained these changes with the decrease in the abundance of the genus *Cylindrospermosis* in the month of August. This implies that phosphorus is the primary nutrient limiting the productivity of this phytoplankton genus since its abundance increased in July and September (Hecky and Kilham, 1988; Howarth and Marino, 2006; Figure 2A). Likewise, the findings of this study are consistent with the behavior of the PSRR phosphorus pathway in July observed in this study (Figure 5D), which implies that the bacterial communities in the water column presented a higher state of P starvation than in the other months, because according to Díaz-Torres et al. (2021), phosphorus was the limiting nutrient in July.

### Sulfur Metabolism

Sulfur is required by all living organisms, including prokaryotes and eukaryotes, as it is essential for the synthesis of amino acids, proteins, and enzymes. Sulfate reduction can occur through assimilatory and dissimilatory pathways (Barton and Fauque, 2009; Kushkevych et al., 2019). In this research, the genes of the bacterial communities associated with the ASR pathway were more abundant than those associated with the DSR pathway (Figure 5B). In a bacterial cell, DSR and ASR are two metabolic processes that metabolize sulfate in distinct ways. The former pathway uses sulfate as a terminal acceptor and is essential for ATP synthesis, with the terminal product of hydrogen sulfide, whereas the latter uses sulfate to form the amino acid cysteine (Barton et al., 2014; Kushkevych, 2016). The ASR is a more complex pathway than the dissimilatory reduction as DSR only requires three enzymes because sulfate-reducing bacteria are strictly anaerobic organisms, whereas seven different enzymes are necessary for the assimilatory pathway as facultative anaerobic microorganisms must operate in both anaerobic and aerobic environments (Kushkevych et al., 2020). During the sampling months, the abundance of genes related to the ASR pathway exhibited a similar pattern to that of the genes involved in the cysteine pathway, indicating that the ASR pathway’s terminal product (cysteine) was produced simultaneously. Cysteine is primarily a protein precursor, but it is also a precursor for vitamins and antioxidants such as glutathione, which is crucial for maintaining the redox homeostasis in many eukaryotes and bacterial cells (Rausch and Wachter, 2005; Helbig et al., 2008). Therefore, it is reasonable to hypothesize that the ASR pathway was the most abundant one in this study, and there were optimal sulfate concentrations for aerobic and anaerobic bacteria to metabolize. While sulfate concentrations were not measured in this study, significant loads of sulfur may enter the lake with wastewater discharge, which facilitates the ASR pathway (Holmer and Storkholm, 2001; de Anda et al., 2019).

## Conclusion

This study provides valuable information on ecological interactions between water quality and bacterial taxonomic and functional composition in a subtropical and hypereutrophic lake during the rainy season. Results showed significant temporal variations over a short period of time, induced by the rainy season, causing high seasonal surface runoff and, therefore, significant changes in the physicochemical (pH, ORP, turbidity, TN, EC, NH_4_^+^ and NO_3_^–^) features of Lake Cajititlán. The most abundant bacterial genera found were *Flavobacterium* and *Pseudomonas*. Bacterial communities displayed a higher relative abundance of genes associated with denitrification, nitrogen fixation, ASR, cysteine, SOX system, and all phosphorus pathways in July, whereas the remaining pathways and months showed similar patterns in abundance as bacterial communities are highly influenced by nutrient loads during the onset of the rainy season (July) and by low DO levels. A limitation of PICRUSt inferences is that they are based on evolutionary modeling of the gene content of known reference genomes. Therefore, the accuracy of any given sample type will depend heavily on the availability of appropriate references. On the other hand, the input data for the standard PICRUSt workflow are partial 16S rRNA gene sequences, and therefore, eukaryotic, or viral contributions to the metagenome will not be predicted. Future research will use shotgun sequencing to comprehensively analyze genes from all organisms present in a sample. This can provide more accurate knowledge of the metabolic mechanisms that may be taking on to allow these organisms to adapt to their environment. Also, these data would allow the further exploration of the functional and taxonomic microbial diversity in freshwater environments and the potential impacts of water quality and climate change (rainy season) on the functioning of these ecosystems.

## Data Availability Statement

The datasets presented in this study can be found in online repositories. The names of the repository/repositories and accession number(s) can be found below: https://www.ncbi.nlm.nih.gov/bioproject/?term=PRJNA626359.

## Author Contributions

OD-T, MG-H, and CS-G conceived the study, designed the methodology, and led the writing of the manuscript. OD-T, JA, AP, and MG-H collected the data. OD-T and CY-M analyzed the data. MG-H, CS-G, OL-M, and AP were responsible for funding acquisition. All authors contributed critically to the drafts and gave final approval for publication.

## Conflict of Interest

The authors declare that the research was conducted in the absence of any commercial or financial relationships that could be construed as a potential conflict of interest.

## Publisher’s Note

All claims expressed in this article are solely those of the authors and do not necessarily represent those of their affiliated organizations, or those of the publisher, the editors and the reviewers. Any product that may be evaluated in this article, or claim that may be made by its manufacturer, is not guaranteed or endorsed by the publisher.

## Footnotes

1
https://www.genome.jp/kegg/ko.html
