# Supplementary figures and images for: Bacterial Dynamics and Their Influence on the Biogeochemical Cycles in a Subtropical Hypereutrophic Lake During the Rainy Season
Source: Front Microbiol. 2022 Apr 5;13:832477. doi: 10.3389/fmicb.2022.832477 (PMC9037096; doi:10.3389/fmicb.2022.832477)

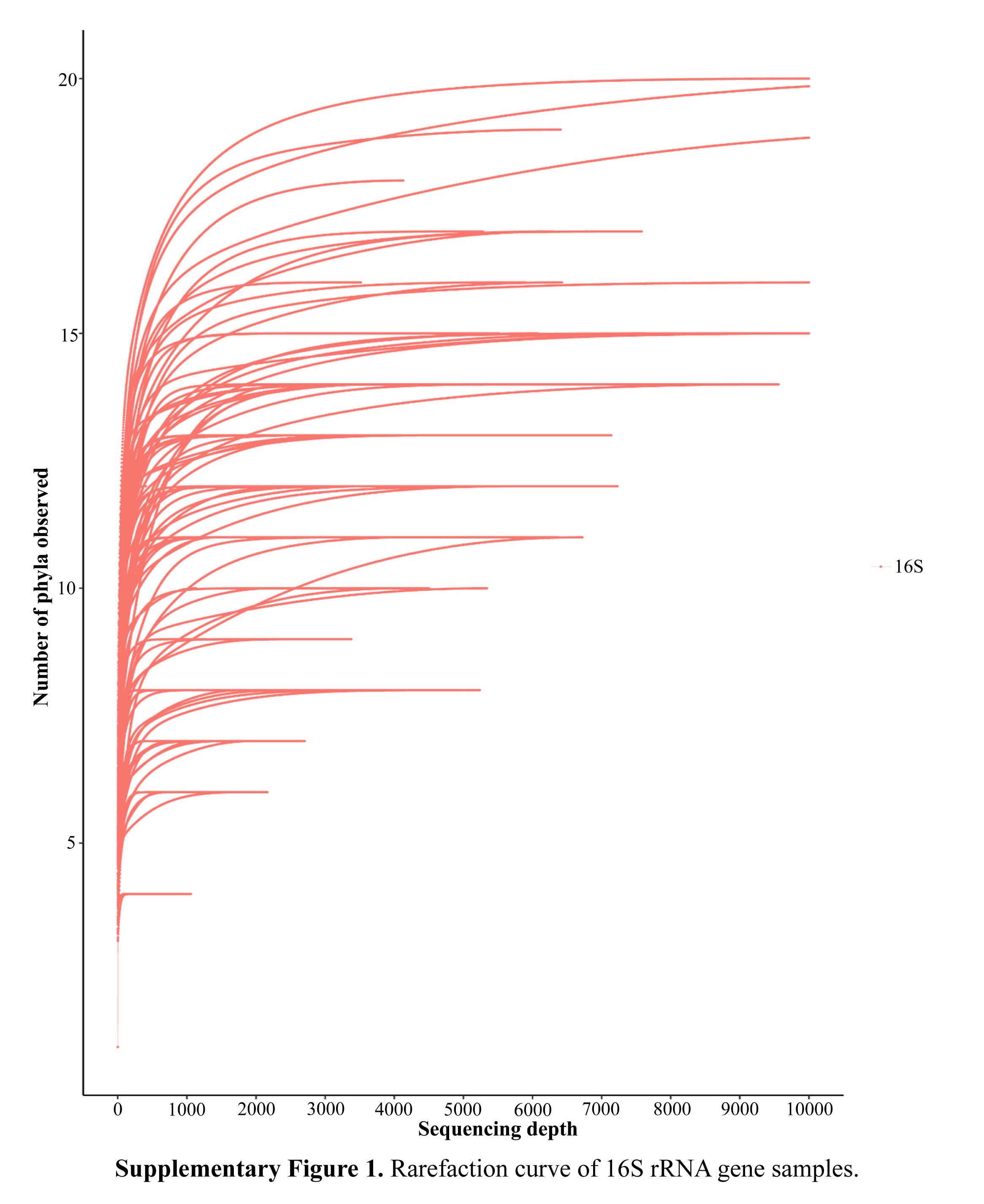

Supplement: Supplementary file 1 [file Image_1.JPEG]

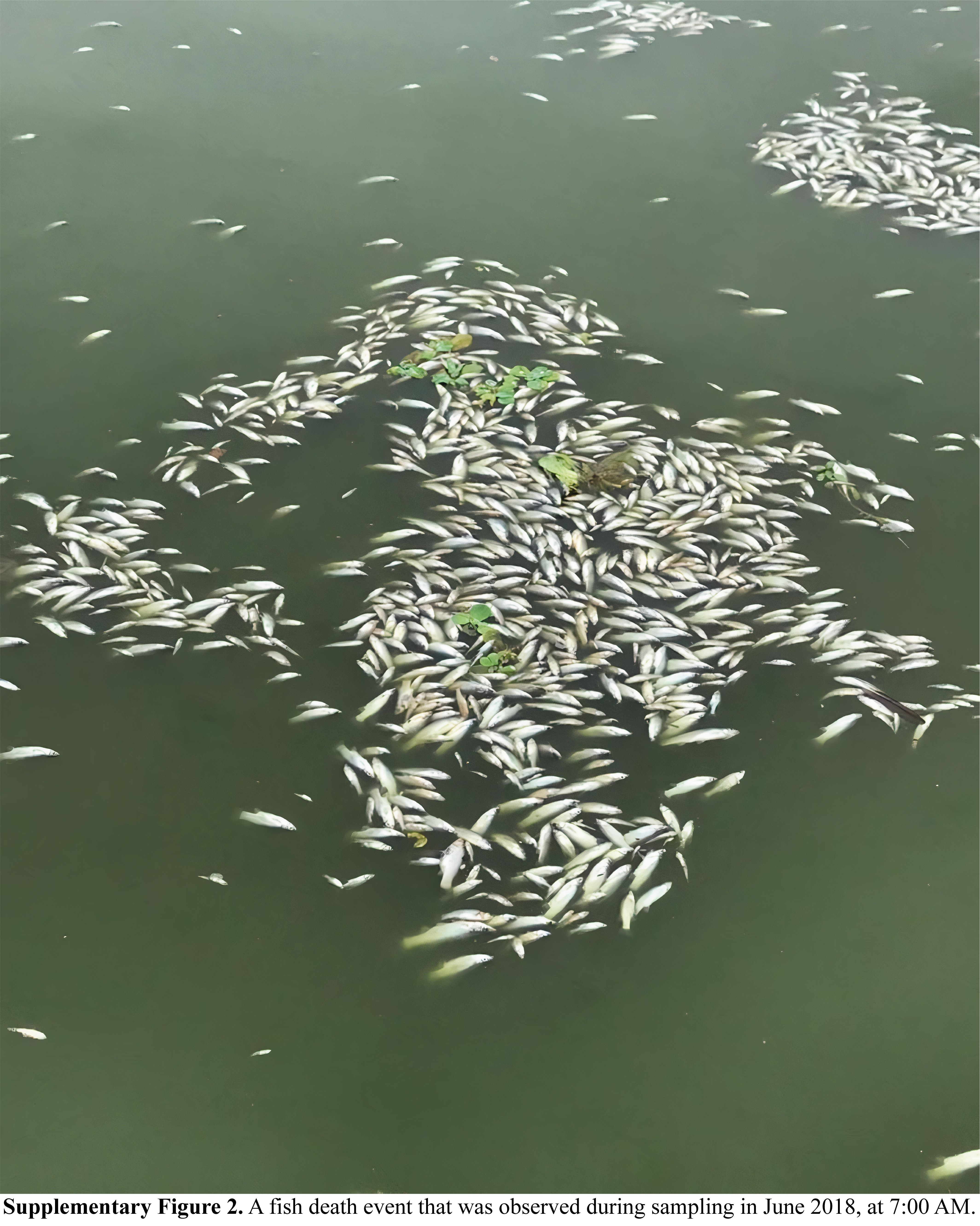

Supplement: Supplementary file 2 [file Image_2.JPEG]
