# Supplementary material for: Bacterial Dynamics and Their Influence on the Biogeochemical Cycles in a Subtropical Hypereutrophic Lake During the Rainy Season
Source: Front Microbiol. 2022 Apr 5;13:832477. doi: 10.3389/fmicb.2022.832477 (PMC9037096; doi:10.3389/fmicb.2022.832477)
Supplement: Supplementary file 3 [file Table_1.DOCX]

Supplementary Tables

**Supplementary Table 1.** Physicochemical and environmental parameters. The asterisk symbol in the parameters denotes statistical significance among months at the p < 0.05 level. Water temperature (WT), dissolved oxygen (DO), pH, electrical conductivity (EC), ammonium (NH_4_^+^), nitrate (NO_3_^-^), turbidity, oxidation-reduction potential (ORP), blue-green algae (BGA-PC), and air temperature (AT).

| **Parameters** | **July**  **Mean ± SD** | **August**  **Mean ± SD** | **September**  **Mean ± SD** |
| --- | --- | --- | --- |
| **DO (mg/L) *** | 2.83 ± 0.61 | 3.25 ± 1.87 | 4.32 ± 1.77 |
| **pH*** | 10 ± 0.05 | 9 ± 0.05 | 9.28 ± 0.19 |
| **WT (°C)** | 24.16 ± 1.55 | 24.73 ± 1.12 | 24.35 ± 0.34 |
| **Turbidity (NTU)*** | 97.03 ± 7.32 | 41.02 ± 7.74 | 68.46 ± 8.52 |
| **ORP (mV)*** | 47.90 ± 12.36 | 66.18 ± 19.30 | 101.78 ± 7.04 |
| **NH_4_^+^ (mg/L) *** | 4.50 ± 0.38 | 4.08 ± 1.46 | 2.49 ± 0.92 |
| **NO_3_^-^ (mg/L) *** | 3.67 ± 0.34 | 2.67 ± 0.56 | 0.87 ± 0.24 |
| **BGA-PC (cell/mL) *** | 275,129 ± 5,551 | 167,662 ± 79,268 | 268,078 ± 3,996 |
| **Chlorophyll-*a* (µg/L) *** | 42.16 ± 5.20 | 42.83 ± 7.40 | 47.40 ± 9.36 |
| **TN (mg/L) *** | 11.46 ± 1.08 | 12.88 ± 2.52 | 9.85 ± 0.63 |
| **TP (mg/L) *** | 1.51 ± 0.06 | 1.52 ± 0.24 | 1.41 ± 0.10 |
| **EC (mS/cm) *** | 1.08 ± 0.003 | 1.21 ± 0.16 | 1.16 ± 0.12 |
| **AT (°C) 2018**  **Max–Min** | 22.37 ± 0.90  24.25-20.75 | 21.63 ± 1.70  24-15.5 | 22.19 ± 0.49  23-21 |
| **Evaporation (mm) 2018**  **Max-Min** | 5.3 ± 1.91  12-2.1 | 4.77 ± 1.71  10.1-0.9 | 4.22 ± 1.76  12.1-0.9 |
| **Precipitation (mm) 2018**  **Max–Min** | 5.77 ± 10.51  40–0 | 3.04 ± 5.35  24–0 | 3.97 ± 10.10  53.2–0 |
| **AT (°C) 1998–2018**  **Max–Min** | 19.88 ± 7.33  26.50-13.26 | 19.47 ± 7.43  26.12-12.83 | 19.33 ± 7.19  25.82-12.85 |
| **Evaporation (mm) 1998–2018** | 5.39 ± 2.29 | 4.90 ± 2.01 | 4.49 ± 2.07 |
| **Precipitation (mm) 1998–2018** | 7.60 ± 13.29 | 5.93 ± 10.89 | 5.47 ± 10.84 |

**Supplementary Table 2.** Pairwise dissimilarity tests of bacterial composition for the different months using ADONIS. The numbers outside the parentheses are “R^2^.” P-values are in parentheses. Bold values denote statistical significance at the p < 0.05 level.

| **Bacterial composition** | **Adonis statistical comparisons** |
| --- | --- |
| All | **0.001(0.001)** |
| Jul vs. Aug | **0.003(0.007)** |
| Jul vs. Sep | **0.001(0.001)** |
| Aug vs. Sep | **0.007(0.001)** |

**Supplementary Table 3.** Pairwise dissimilarity tests of bacterial composition for the different sampling sites using ADONIS. The numbers outside the parentheses are “R^2^.” P-values are in parentheses.

| **Bacterial composition** | **Adonis statistical comparisons** |
| --- | --- |
| All | 0.263(0.410) |
| CEA-1 vs. CEA-2 | 0.700(0.702) |
| CEA-1 vs. CEA-3 | 0.524(0.285) |
| CEA-1 vs. CEA-4 | 0.223(0.182) |
| CEA-1 vs. CEA-5 | 0.234(0.157) |
| CEA-2 vs. CEA-3 | 0.929(0.891) |
| CEA-2 vs. CEA-4 | 0.552(0.808) |
| CEA-2 vs. CEA-5 | 0.245(0.233) |
| CEA-3 vs. CEA-4 | 0.232(0.343) |
| CEA-3 vs. CEA-5 | 0.176(0.107) |
| CEA-4 vs. CEA-5 | 0.788(0.674) |

**Supplementary Table 4.** Pairwise dissimilarity tests of functional composition for the different months using ADONIS. The numbers outside the parentheses are “R^2^.” P-values are in parenthes^i^s. Bold values denote statistical significance at the p < 0.05 level.

| **Adonis statistical comparisons** | | | | | |
| --- | --- | --- | --- | --- | --- |
| **Functional composition** | **All** | **N** | **P** | **C** | **S** |
| All | **0.003(0.008)** | **0.001(0.001)** | **0.001(0.001)** | 0.552(0.067) | **0.022(0.045)** |
| Jul vs. Aug | **0.001(0.001)** | **0.001(0.001)** | **0.001(0.001)** | 0.315(0.080) | **0.02(0.089)** |
| Jul vs. Sep | **0.012(0.002)** | **0.001(0.001)** | **0.001(0.001)** | 0.054(0.084) | 0.06(0.088) |
| Aug vs. Sep | **0.161(0.022)** | 0.289(0.134) | 0.41(0.498) | 0.082(0.322) | 0.138(0.121) |

**Supplementary Table 5.** Pairwise dissimilarity tests of functional composition for the different sampling sites using ADONIS. The numbers outside the parentheses are “R^2^.” P-values are in parenthesis. Bold values denote statistical significance at the p < 0.05 level.

| **Adonis statistical comparisons** | | | | | |
| --- | --- | --- | --- | --- | --- |
| **Functional composition** | **All** | **N** | **P** | **C** | **S** |
| All | 0.161(0.268) | 0.372(0.635) | 0.470(0.924) | 0.171(0.254) | 0.389(0.645) |
| CEA-1 vs. CEA-2 | 0.572(0.551) | 0.521(0.807) | 0.852(0.285) | 0.108(0.117) | 0.264(0.222) |
| CEA-1 vs. CEA-3 | 0.662(0.605) | 0.777(0.661) | 0.371(0.054) | 0.346(0.498) | 0.391(0.283) |
| CEA-1 vs. CEA-4 | 0.607(0.516) | 0.255(0.179) | 0.714(0.266) | 0.967(0.948) | 0.312(0.695) |
| CEA-1 vs. CEA-5 | 0.816(0.762) | 0.828(0.729) | 0.615(0.289) | 0.557(0.723) | 0.38(0.648) |
| CEA-2 vs. CEA-3 | 0.908(0.892) | 0.85(0.707) | 0.675(0.672) | 0.06(0.255) | 0.693(0.866) |
| CEA-2 vs. CEA-4 | 0.528(0.639) | 0.313(0.376) | 0.875(0.900) | **0.042(0.007)** | 0.062(0.824) |
| CEA-2 vs. CEA-5 | 0.798(0.871) | 0.592(0.747) | 0.953(0.817) | 0.721(0.530) | 0.391(0.529) |
| CEA-3 vs. CEA-4 | 0.55(0.465) | 0.263(0.128) | 0.628(0.595) | 0.136(0.174) | 0.266(0.507) |
| CEA-3 vs. CEA-5 | 0.834(0.800) | 0.706(0.333) | 0.977(0.602) | 0.129(.479) | 0.803(0.484) |
| CEA-4 vs. CEA-5 | 0.726(0.950) | 0.509(0.886) | 0.891(0.923) | 0.514(0.422) | 0.216(0.807) |

**Supplementary Table 6.** Redundancy analysis (RDA) of bacterial communities. Eigenvalues, proportion explained, and cumulative proportion by eight RDA axes are shown.

| **Component** | **Eigenvalue** | **Proportion explained** | **Cumulative proportion** |
| --- | --- | --- | --- |
| RD1 | 2.86 | 0.289 | 0.289 |
| RD2 | 1.47 | 0.150 | 0.439 |
| RD3 | 2.81 | 0.283 | 0.722 |
| RD4 | 0.88 | 0.092 | 0.814 |
| RD5 | 0.73 | 0.078 | 0.892 |
| RD6 | 0.58 | 0.062 | 0.954 |
| RD7 | 0.28 | 0.033 | 0.987 |
| RD8 | 0.10 | 0.013 | 1 |

# Supplementary Table 7. Eigenvalues of the physicochemical variables in the RDA of bacterial communities. Water temperature (WT), dissolved oxygen (DO), pH, electrical conductivity (EC), ammonium (NH_4_^+^), nitrate (NO_3_^-^), turbidity, oxidation-reduction potential (ORP), blue-green algae (BGA-PC).

| **Variable** | **RDA1** | **RDA2** | **RDA3** | **RDA4** |
| --- | --- | --- | --- | --- |
| DO | 0.209 | **0.417** | -0.051 | 0.116 |
| pH | **-0.632** | -0.326 | **-0.466** | 0.013 |
| WT | -0.315 | 0.314 | -0.106 | **-0.378** |
| Turbidity | -0.444 | **-0.486** | -0.289 | 0.037 |
| ORP | **-0.776** | 0.075 | -0.093 | 0.185 |
| NH_4_^+^ | **-0.771** | 0.036 | -0.046 | 0.094 |
| NO_3_^-^ | **-0.927** | 0.070 | -0.105 | 0.193 |
| BGA-PC | 0.079 | -0.190 | -0.269 | -0.017 |
| Chlorophyll-*a* | 0.213 | -0.123 | 0.077 | -0.311 |
| EC | 0.189 | 0.089 | 0.225 | **-0.633** |
| TN | -0.509 | 0.167 | **0.426** | -0.060 |
| TP | -0.424 | 0.105 | -0.150 | 0.033 |

**Supplementary Table 8.** Redundancy analysis (RDA) of functional composition. Eigenvalues, proportion explained, and cumulative proportion by 12 RDA axes are shown.

| **Component** | **Eigenvalue** | **Proportion explained** | **Cumulative proportion** |
| --- | --- | --- | --- |
| RD1 | 8.640 | 0.890 | 0.890 |
| RD2 | 0.351 | 0.036 | 0.926 |
| RD3 | 0.283 | 0.029 | 0.955 |
| RD4 | 0.159 | 0.016 | 0.971 |
| RD5 | 0.098 | 0.010 | 0.981 |
| RD6 | 0.062 | 0.006 | 0.987 |
| RD7 | 0.051 | 0.005 | 0.992 |
| RD8 | 0.023 | 0.002 | 0.994 |
| RD9 | 0.020 | 0.002 | 0.996 |
| RD10 | 0.020 | 0.002 | 0.998 |
| RD11 | 0.010 | 0.001 | 0.999 |
| RD12 | 0.010 | 0.001 | 1 |

**Supplementary Table 9.** Eigenvalues of the physicochemical variables in the RDA of functional composition. Water temperature (WT), dissolved oxygen (DO), pH, electrical conductivity (EC), ammonium (NH_4_^+^), nitrate (NO_3_^-^), turbidity, oxidation-reduction potential (ORP), blue-green algae (BGA-PC).

| **Variable** | **RDA1** | **RDA2** | **RDA3** | **RDA4** |
| --- | --- | --- | --- | --- |
| DO | 0.304 | 0.211 | 0.220 | 0.211 |
| pH | **-0.933** | -0.010 | -0.053 | -0.054 |
| WT | 0.061 | -0.189 | 0.508 | -0.319 |
| Turbidity | **-0.833** | **0.330** | -0.172 | 0.153 |
| ORP | **-0.811** | -0.070 | 0.305 | 0.073 |
| NH_4_^+^ | -0.448 | -0.163 | **0.547** | -0.470 |
| NO_3_^-^ | -0.741 | -0.136 | **0.497** | -0.106 |
| BGA-PC | -0.415 | 0.157 | -0.361 | 0.479 |
| Chlorophyll-*a* | 0.182 | -0.256 | -0.011 | **-0.612** |
| EC | 0.413 | -0.273 | -0.156 | 0.123 |
| TN | -0.026 | 0.234 | 0.434 | -0.126 |
| TP | -0.131 | -0.249 | 0.467 | -0.480 |
